# Neuropsychiatric Disorders Associated With Vitamin B12 Deficiency: An Autobiographical Case Report

**DOI:** 10.7759/cureus.21476

**Published:** 2022-01-21

**Authors:** Ahmed Badar

**Affiliations:** 1 Department of Physiology, College of Medicine, Imam Abdulrahman Bin Faisal University, Dammam, SAU

**Keywords:** neuropathy, neurotransmitters, myelin, neuropsychiatric disorders, vitamin b12 deficiency

## Abstract

Vitamin B12 is an essential vitamin. Among its many functions is its crucial role as a coenzyme in a step of normal synthesis of myelin. Likewise, it is a vital coenzyme in the synthesis of neurotransmitters. Therefore, it is no surprise that vitamin B12 deficiency is linked to a spectrum of neuropsychiatric disorders. Most physicians can quickly pick up typical pernicious anemia, but very few remember the association of vitamin B12 with neuropsychiatric disorders. In this case report, the author presents his own experience of neuropsychiatric disorder (presenting as carpal tunnel syndrome along with anxiety) related to vitamin B12 deficiency with a strong recommendation to include vitamin B12 in the initial set of laboratory investigations suggested for patients over 40 years of age presenting with neuropsychiatric disorders.

## Introduction

Myelin is required for neuronal protection as well as normal transmission of impulses in the nerves. The one-carbon cycle is vital for the synthesis of not only myelin but also DNA, RNA, membrane phospholipids, and neurotransmitters. Several B-complex vitamins including vitamin B12, vitamin B6, vitamin B2, and folate are required for the one-carbon cycle [1]. Vitamin B12 is crucial for the one-carbon cycle as it acts as a coenzyme for the synthesis of methionine, which, in turn, donates the methyl group required for methylation, one of the most important steps in the cycle. In addition, as methionine is formed from homocysteine, if methionine formation is stopped, abnormal amounts of homocysteine accumulate, leading to cognitive disturbances as well as damage to the neurons [2].

Vitamin B12 is an essential coenzyme for the conversion of methylmalonyl-CoA to succinyl CoA, which is a basic requirement for myelin synthesis and stabilization. In the absence of cobalamin, methylmalonyl-CoA forms methylmalonic acid (MMA) that forms abnormal fatty acids, leading to abnormal myelination or demyelination [3].

For the aforementioned reasons, adequate amounts of vitamin B12 are crucial for normal neuropsychiatric health. In this case report, the author shares his experience of peripheral neuropathy and psychological issues related to vitamin B12 deficiency and improvement with treatment.

## Case presentation

How it started (presenting complaints)

I started experiencing dull pain in both the hands and wrists in June 2019. The discomfort in the wrists was present all the time and gradually built up. Pain in the hands could not be localized to specific locations and increased on holding or twisting anything. At times there was numbness, at others a feeling like locking of fingers. I considered arthritis, neuropathy, and bilateral carpal tunnel syndrome (CTS). The pain was at least twice more on the left side (5 on a scale of 1-10 on the left side). A few days later, I started experiencing shooting pain in my left arm and left pectoral region. Often the pain in the arm radiated from the ulnar side of the left arm right up to the wrist. The pain in the chest was never retrosternal, mostly started at rest, stayed for a variable amount of time (2-10 minutes), did not exacerbate with exertion, and was frequently associated with numbness of left chest muscles. At that time, my age was 55 years and my body mass index (BMI) was 27.5 kg/m^2^. I was not a smoker, a diabetic, and did not drink alcohol. I started getting very anxious and panicked due to my previous experiences.

History

It was not unusual for me to have psychological and physical symptoms (anxiety, catastrophizing, and somatization) in times of stress since the days of my medical school. In 1991, I opted for teaching (and research) as my career and stopped direct interaction with patients. I had a car accident in June 1994 when I was 30 years of age. I was stuck upside down for a few minutes with all my 85 kg weight on my neck. I was declared fit and cleared to go home with a few minor scratches after the rescue. A few weeks later, I started my studies for a master’s degree that involved a lot of writing and working on polygraphs as well as desktop PCs of that era. Very soon, I started noticing pain in my writing (right) hand while holding a pen, writing, or typing, mainly in the ring and little fingers. Very soon, I got very anxious, “catastrophized” this symptom, and started getting vertigo and panic attacks while experiencing pain. Scans of the cervical spine showed minimal herniation at C5-6. A neck collar and physiotherapy relieved the pain; however, the pain kept recurring on lifting weight, jumping, typing for a long time, or jerks during driving. I learned to live with occasional pain and largely forgot about it. In June 2003, I suffered a fracture of the right acetabulum (with posterior dislocation of the femur head) in another road traffic accident. It was operated upon, and plating was performed. In 2010, I was diagnosed with duodenal ulcers on endoscopy after a prolonged spell of acid peptic disease. It responded to *Helicobacter pylori* therapy, recurred a couple of times, but eventually settled. This was followed by gastroesophageal reflux in 2012 that I managed with lifestyle modifications. In 2016, I experienced severe left shoulder pain, and the scans showed a partial tear in the rotator cuff which responded to analgesics and physiotherapy. I have managed to live with it with lifestyle changes. I have been hypertensive since 2012 and control it with valsartan, walking, and diet.

Consultations and investigations

I was lucky to have good students and friends in all the relevant disciplines. A cardiologist was the first one to examine, run a treadmill stress test, followed by an echo, and assured me that the left arm and chest symptoms were not cardiac and that the pain was not ischemic. This was followed by a visit to an orthopedic surgeon who examined and informed me that it was CTS and the chest and arm pains are due to an old tear in the rotator cuff tendon and sympathetic contraction of muscles when the shooting pain starts from the tear. Analgesics and hot/cold fomentation would reduce the problem but, eventually, the tendon would need repair. The suggested treatment did not help much except for temporary relief. The next was a neurosurgeon who listened to my history and obtained scans of the cervical spine. Scans showed minimal herniation at C5-6 (like 1994) and age-related changes. He assured me that at that time surgery was not required and the best option was physiotherapy. By the sevenths session of physiotherapy, I realized that there was no advantage at all and the symptoms were progressing.

Next, I consulted a neurologist colleague. She arranged for urgent nerve conduction studies and prescribed some lab tests for which I gave blood without even noticing them as I had lost my faith in examinations and investigations over those few weeks of continuous pain and associated anxiety. The next day she forwarded me the report of nerve conduction study that concluded: “bilateral mild distal median neuropathy at wrist consistent with a diagnosis of bilateral mild CTS” (Tables 1, 2). She did not immediately answer my query of “what next?,” and I lost hope and decided to think of another approach. The very next morning she called me and said, “Dr. Ahmed you have very low levels of vitamin B12 (<148 pg/mL). All the other tests are normal and there is no evidence of megaloblastic anemia. Please go to the pharmacy, I have prescribed injection vitamin B12 for you. Start today, one daily for six days, followed by once weekly for four weeks and then once monthly for the rest of your life. May Allah give you a long life.”

**Table 1:** Motor nerve conduction study.

Nerve and site	Latency	Amplitude	Segment	Latency difference	Distance	Conduction velocity
Median. L						
Wrist	4.2 ms	12.0 mV	Abductor pollicis brevis-Wrist	4.2 ms	80 mm	m/s
Elbow	8.7 ms	10.9 mV	Wrist-Elbow	4.5 ms	230 mm	51 m/s
Ulnar. L						
Wrist	3.3 ms	10.8 mV	Abductor digiti minimi (manus)-Wrist	3.9 ms	80 mm	m/s
Below elbow	6.9 ms	8.3 mV	Wrist-Below elbow	3.0 ms	220 mm	73 m/s
Above elbow	7.7 ms	11.4 mV	Below elbow-Above elbow	0.8 ms	100 mm	125 m/s
Radial. L						
Forearm	2.3 ms	3.5 mV	Extensor indicis proprius-Forearm	2.3 ms	mm	m/s
Lateral brachium	5.5 ms	4.1 mV	Forearm-Lateral brachium	3.1 ms	mm	m/s
Axillary. L						
Supraclavicular fossa	4.3 ms	14.6 mV	Deltoid-Supraclavicular fossa	4.3 ms	mm	m/s
Median. R						
Wrist	4.0 ms	12.1 mV	Abductor pollicis brevis-Wrist	4.0 ms	80 mm	m/s
Elbow	8.2 ms	12.0 mV	Wrist-Elbow	4.2 ms	220 mm	52 m/s
Ulnar. R						
Wrist	3.3 ms	6.9 mV	Abductor digiti minimi (manus)-Wrist	3.6 ms	80 mm	m/s
Below elbow	6.8 ms	7.0 mV	Wrist-Below elbow	3.2 ms	230 mm	72 m/s
Above elbow	8.2 ms	7.0 mV	Below elbow-Above elbow	1.4 ms	100 mm	71 m/s
Radial. R						
Forearm	1.8 ms	2.3 mV	Extensor indicis proprius-Forearm	1.8 ms	80 mm	m/s
Lateral brachium	5.2 ms	1.5 mV	Forearm-Lateral brachium	3.4 ms	240 mm	71 m/s
Axillary. R						
Supraclavicular fossa	4.3 ms	10.8 mV	Deltoid-Supraclavicular fossa	4.3 ms	mm	m/s

**Table 2:** Sensory nerve conduction and F-wave studies.

Sensory nerve conduction										
Nerve and site	Onset latency		Peak latency		Amplitude		Segment	Latency difference	Distance	Conduction velocity
Median. L										
	ms		ms		µV		Digit II (index finger)-Wrist	3.2 ms	140 mm	44 m/s
Wrist	3.2 ms		3.9 ms		48 µV			ms	mm	m/s
Ulnar. L										
Wrist	2.4 ms		3.3 ms		54 µV		Digit V (little finger)-Wrist	2.4 ms	140 mm	58 m/s
Median. R										
	ms		ms		µV		Digit II (index finger)-Wrist	2.7 ms	140 mm	52 m/s
Wrist	2.7 ms		3.6 ms		26 µV			ms	mm	m/s
Ulnar. R										
Wrist	2.3 ms		3.3 ms		43 µV		Digit V (little finger)-Wrist	2.3 ms	130 mm	57 m/s
F-wave studies										
Nerve		M-latency		F-latency						
Median. L		8.7		28.1						
Ulnar. L		7.7		28.5						
Median. R		4.0		30.0						
Ulnar. R		8.2		31.4						

Prognosis and follow-up

All the symptoms including pain, numbness, and anxiety improved by the fourth injection. They completely disappeared in two weeks. They have not recurred in the last 25 months. I regularly inject myself with 1,000 µg intramuscularly monthly in the quadriceps. A few times when I delayed my monthly injection my fingers started feeling a little “different.” This might be my “anxious” mind as I still do not find evidence of such an acute impact of borderline deficiency except for myself. So far, we have been unable to trace the cause (gastric atrophy or any other) of vitamin B12 deficiency, and it is currently maintained within the normal range.

## Discussion

Vitamin B12 deficiency is mostly detected in patients aged more than 40 years. In this age group, it has been associated with a wide spectrum of neuropsychiatric diseases. Symptoms related to vitamin B12 deficiency can be diverse and vary from neurologic to psychiatric. The current evidence points to muscle wasting/weakness, myelopathy, neuropathy, and gait disorders as neurological manifestations. The psychiatric disorders linked to vitamin B12 deficiency range from behavioral disturbances and cognition problems to dementia [3].

Peripheral neuropathy is the most common presentation of vitamin B12 deficiency. Depending upon the type of nerve involved, it may present as pain, numbness, tingling, loss of sensation, decreased motor activity, or decreased muscle mass. Early diagnosis and treatment might lead to full or partial recovery and limit further damage [4]. The proposed mechanisms include hypomethylation, phospholipid metabolism, and neurotoxic effects of homocysteine accumulation [5]. Nerve conduction studies on patients with confirmed vitamin B12-associated peripheral neuropathy have demonstrated severe impairment of sensory nerve conduction reflecting demyelinating disorder. Studies suggest that lack of vitamin B12 may lead to primary sensory demyelinating neuropathy [6]. A study testing somatosensory central conduction time (CCT) in patients with vitamin B12 deficiency reported a striking increase in CCT in absence of any upper motor neuron lesion sign. The study reported that the decrease in conduction velocity occurred in the posterior columns but was normal at the thalamocortical level [7].

The risk of peripheral neuropathy is profoundly more in diabetic patients, especially those on metformin [8], and patients with atrophic gastritis [9]. A recent systematic review of 32 studies reported that neuropathy was associated with lowered vitamin B12 levels (pooled estimate (95% confidence interval) = 1.51 (1.23-1.84)) based on the cut-off value of ≥205 ng/L. The study reported an association with different types of neuropathies among which diabetes was most significant. It recommended screening for vitamin B12 deficiency for patients at risk for neuropathy and those with neuropathy [10].

There is evidence of frequent association of CTS with vitamin B12 deficiency. Preventing vitamin B12 deficiency may help alleviate complaints related to CTS with less invasive treatment modalities [11].

Vitamin B12 deficiency may also cause spinal cord lesion or subacute combined degeneration where a demyelination process leads to the decay of the myelin sheath in the dorsal and lateral columns [12]. It is a progressive degenerative disease of the spinal cord that may involve some cranial nerves as well. Early treatment with vitamin B12 leads to excellent improvement in symptoms while a delay leads to incomplete recovery [13]. Electrophysiological studies in patients with subacute combined degeneration of the cord showed features of axonal neuropathy and evidence of prominent focal proximal conduction block in several nerves, which markedly improved after treatment with cyanocobalamin [14].

Visual pathways are very vulnerable to vitamin B12 deficiency. The visual evoked potential is often found prolonged in patients with vitamin B12 deficiency neurological syndrome even if they are otherwise asymptomatic. It has been shown to improve after treatment with vitamin B12 [15].

There is evidence of faster age-related changes in the brain in the presence of vitamin B12 deficiency [16]. Studies have associated Alzheimer’s disease, Parkinson’s disease, various conditions causing dementia, and even multiple sclerosis with vitamin B12 deficiency [17]. However, for most of these, there is no evidence of a causal relationship and the findings remain controversial.

Several psychiatric problems have been associated with vitamin B12 deficiency that include but are not limited to depression, delusions, cognitive changes, dementia, mania, psychosis, and occasional suicidal thoughts [3]. Proposed mechanisms include impairment of neurotransmitter synthesis and increased amount of homocysteine as well as MMA [5]. Vitamin B12 therapy may lead to improvement in dementia in some patients.

A recent systematic review concluded that deficiency of vitamin B12 is associated with a higher risk of developing depression; however, it did not find any evidence of improvement in depressive symptoms by administration of vitamin B12 [18]. There are case reports of acute psychosis as a symptom of vitamin B12 deficiency as well as of acute onset of mania and gait disorder that responded well to vitamin B12 therapy [19]. A recent clinical trial concluded that vitamin B12 deficiency is an overlooked cause of gait disorders in elderly patients with psychiatric problems, and vitamin B12 therapy reduced the risk of falls in them [20].

## Conclusions

Vitamin B12 deficiency is a frequently ignored cause of neuropathy and psychiatric disorders. It is frequently found in older age groups, diabetes patients, and patients with a history of acid peptic disease. It can cause neuropsychiatric disorders or worsen them. Vitamin B12 therapy results in a reversal of neuropsychiatric symptoms or improvement in most patients. It is recommended to keep vitamin B12 in mind while ordering the first set of investigations in patients aged 40 years or more presenting with neuropsychiatric disorders even if there is no hematological manifestation.
